# Impact of partial pressure of arterial oxygen and radiologic findings on postoperative acute exacerbation of idiopathic interstitial pneumonia in patients with lung cancer

**DOI:** 10.1007/s00595-023-02711-y

**Published:** 2023-06-06

**Authors:** Yoko Azuma, Susumu Sakamoto, Sakae Homma, Takashi Sakai, Satoshi Koezuka, Megumi Kamemura, Naobumi Tochigi, Akira Iyoda

**Affiliations:** 1 Division of Chest Surgery, Department of Surgery, Toho University School of Medicine, 6-11-1 Omori-Nishi, Ota-Ku, Tokyo, 143-8541 Japan; 2 Department of Respiratory Medicine, Toho University School of Medicine, 6-11-1 Omori-Nishi, Ota-Ku, Tokyo, 143-8541 Japan; 3 Department of Surgical Pathology, Toho University School of Medicine, 6-11-1 Omori-Nishi, Ota-Ku, Tokyo, 143-8541 Japan

**Keywords:** Lung cancer, Idiopathic interstitial lung disease, Postoperative acute exacerbation

## Abstract

**Purpose:**

To establish accurate diagnostic criteria and predictors of treatment response for postoperative acute exacerbation (AE) in patients with lung cancer and idiopathic interstitial pneumonia (IIP).

**Methods:**

Among 93 patients with IIP who underwent surgery for lung cancer, suspected postoperative AE developed in 20 (21.5%). Patients were divided into a progressive AE group, comprising patients with bilateral alveolar opacities and decreasing PaO_2_ ≥ 10 mmHg (*n* = 5); an incipient AE group, comprising patients with unilateral alveolar opacities and decreasing PaO_2_ ≥ 10 mmHg (*n* = 10); and an indeterminate AE group, comprising patients with alveolar opacities but decreasing PaO_2_ < 10 mmHg (*n* = 5).

**Results:**

The progressive AE group had significantly higher 90-day mortality (80%) than the incipient AE group (10%, *P* = 0.017) or the indeterminate AE group (0%, *P* = 0.048). Bilateral opacities may indicate advanced AE and poor prognosis, whereas unilateral opacities may indicate an early stage of AE and a good prognosis. PaO_2_ < 10 mmHg may indicate conditions other than AE.

**Conclusions:**

In patients with lung cancer and IIP, decreasing PaO_2_ and HRCT findings may allow for the initiation of rapid and accurate treatment strategies for postoperative AE.

**Supplementary Information:**

The online version contains supplementary material available at 10.1007/s00595-023-02711-y.

## Introduction

Patients with interstitial lung disease (ILD) are at increased risk of lung cancer, and fatal complications such as acute exacerbation (AE) of ILD associated with treatments for lung cancer are a serious concern [1]. A large multi-institutional cohort study of 1763 patients with ILD who had undergone lung cancer surgery identified an incidence of postoperative AE of 9.3% and a mortality rate of 43.9% [2]. Among the classifications of ILD, idiopathic interstitial pneumonia (IIP) including idiopathic pulmonary fibrosis (IPF) is the most frequent, and is associated with the highest incidence of postoperative AE [3]. AE of IIP is characterized by the rapid progression of respiratory failure with the appearance of new widespread infiltrates during the chronic course of IPF, a concept identified first in Japan [4]. To date, AE of IPF has been diagnosed according to the international working group report [5] and the Japanese Respiratory Society guidelines [6]. However, these guidelines are not standardized and only the Japanese criteria include decreasing partial pressure of arterial oxygen (PaO_2_) [6]. Although these criteria are based on the pathogenesis of idiopathic AE, they are also being used for AE of IIP, including postoperative AE [5, 6]. In contrast to idiopathic AE, which develops during the chronic course of IIP, postoperative AE occurs in the context of rapid physical and radiological changes triggered by surgical procedures including tracheal intubation, perioperative transfusion, bleeding, pulmonary resection, and increased levels of circulating inflammatory cytokines. Although accurate diagnosis and rapid treatment are required to improve the survival rate of postoperative AE, it is unclear whether the use of criteria for idiopathic AE is appropriate for diagnosing postoperative AE. The present study identified patients with postoperative AE among 93 patients with lung cancer and IIP, and attempted to establish criteria for the accurate diagnosis and prediction of the prognosis of postoperative AE.

## Methods

### Ethical approval

This study was approved by the Ethics Committee of Toho University Omori Medical Center (M20122) on August 28, 2020. Written informed consent or opt-out consent was obtained from all patients.

### Study design and population

Data were collected on patients who had initially undergone pulmonary resection for lung cancer at Toho University Hospital between January, 2004 and February, 2022. IIP was diagnosed in 93 patients based on clinical data, radiographic images, and histopathological findings. In accordance with our previous study [3], the diagnostic classification of IIPs was established by multidisciplinary discussion between pulmonologists, thoracic pathologists, and radiologists, based on the latest 2018 diagnostic criteria by the American Thoracic Society, European Respiratory Society, Japanese Respiratory Society, and the Latin American Thoracic Society. [7]

**Fig. 1:**
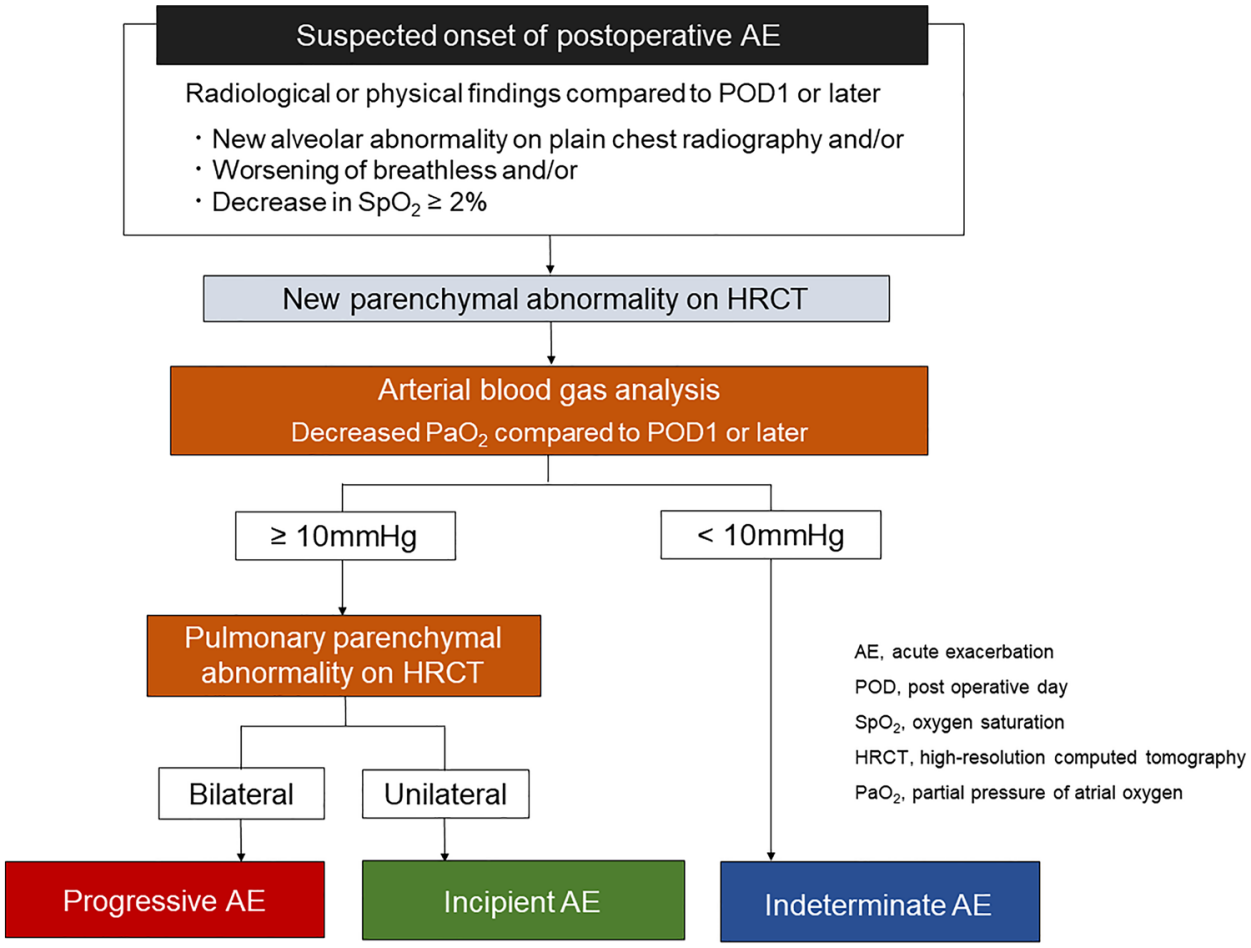
Schema of diagnostic and classification criteria

Figure 1 shows the diagnoses and classification of patients with suspected postoperative AE. AE was defined according to the Japanese Respiratory Society guidelines 2022, [6] and the threshold for decreasing PaO_2_ was set as 10 mmHg. The postoperative period was defined as the first month following surgery.

AE was suspected if any of the following radiological or physical changes were identified from postoperative day (POD) or later: (i) new abnormal opacity on plain chest radiography; (ii) worsening of breathlessness; and/or (iii) > 2% decrease in peripheral artery oxygen saturation at rest or on exertion. Arterial blood gas analysis (BGA) was performed for patients with new parenchymal abnormalities on high-resolution computed tomography (HRCT), excluding shadows around the staple line. Patients were divided into three groups based on new parenchymal abnormalities on HRCT and BGA findings: (i) progressive AE group (*n* = 5): patients with bilateral alveolar opacities and decreasing PaO_2_ ≥ 10 mmHg, (ii) incipient AE group (*n* = 10): patients with unilateral alveolar opacities and decreasing PaO_2_ ≥ 10 mmHg; and (iii) indeterminate AE group (*n* = 5): patients without alveolar opacities but decreasing PaO_2_ < 10 mmHg. Patients with deterioration attributed to cardiac failure or fluid overload were excluded. PaO_2_ values were compared with those on POD 1 or later under similar conditions. We evaluated PaO_2_ values in principle in room air. For patients on home oxygen therapy, we assessed the PaO_2_ values under oxygen administration.

Table 1 shows the baseline characteristics of all patients eligible for this study. The mean age of the patients was 69.5 years and the majority were men (*n* = 18, 90.0%). The mean duration of tobacco smoking was 48.3 pack-years. The mean preoperative predicted forced vital capacity (FVC%pred) was 93.7%. The mean predicted diffusing capacity of the lungs for carbon monoxide (%DL_CO_) was 70.8%. The mean predicted forced expiratory ventilation in 1 s was 98.1%. The most common HRCT pattern of IIP was UIP or probable UIP (*n* = 17, 85.0%). Seven (35.0%) patients were diagnosed with pathological stage 2 and 3 lung cancer and 18 (90.0%) patients underwent lobectomy.

**Table 1:** Baseline characteristics of the patients eligible for this study

Age, years	69.5 ± 5.8
Male sex	18 (90.0)
Tobacco, pack-years	48.3 ± 24.4
KL-6 (U/mL)	737.1 ± 396.0
*Pulmonary function*	
FVC%pred (%)	93.7 ± 17.6
DL_CO_%pred (%)	70.8 ± 15.1
%FEV_1_ pred (%)	98.1 ± 19.8
*HRCT pattern*	
UIP or probable UIP/other	17 (85.0)/ 3 (15.0)
*Comorbidities*	
COPD	9 (45.0)
Coronary artery disease	3 (15.0)
*Pathologic staging*	
I/ II/ III/ IVA	5 (25.0)/ 7 (35.0)/ 7 (35.0)/ 1 (5.0)
*Surgical procedure*	
Bilobectomy/ lobectomy/ partial resection	1 (5.0)/ 18 (90.0)/ 1 (5.0)

Data are presented as *n* (%) or mean ± SD. KL-6, Krebs von Den Lungen-6; FVC%pred, percentage of predicted forced vital capacity, DL_CO_%pred: percentage of predicted diffusing capacity of the lungs for carbon monoxide, %FEV_1_pred: percentage of predicted forced expiratory ventilation in 1 s (%); *PaO*_*2*_ arterial oxygen partial pressure, *FiO*_*2*_ fraction of inspired oxygen, *HRCT* high-resolution computed tomography, *UIP* usual interstitial pneumonia, *COPD* chronic obstructive pulmonary disease

### Statistical analyses

Data are presented as medians with interquartile ranges or mean ± standard deviation (SD). Categorical variables are expressed as percentages. For comparisons between two groups, Fisher’s exact test was used for variables with a normal distribution and the Dunnett test was used for variables with a non-normal distribution. P-values less than 0.05 were considered significant. Overall survival (OS) was defined as the time from the date of surgery until the date of the last follow-up for living patients, or until death. The Kaplan–Meier method was used to plot survival curves. Univariate analysis was performed using the log-rank test. Multivariate analysis was performed using the Cox proportional hazards. JMP version 17.0 (SAS Institute Inc., Cary, NC, USA) was used for all statistical analyses.

## Results

### Baseline characteristics and findings at the time of suspected AE onsetsec2

**Fig. 2:**
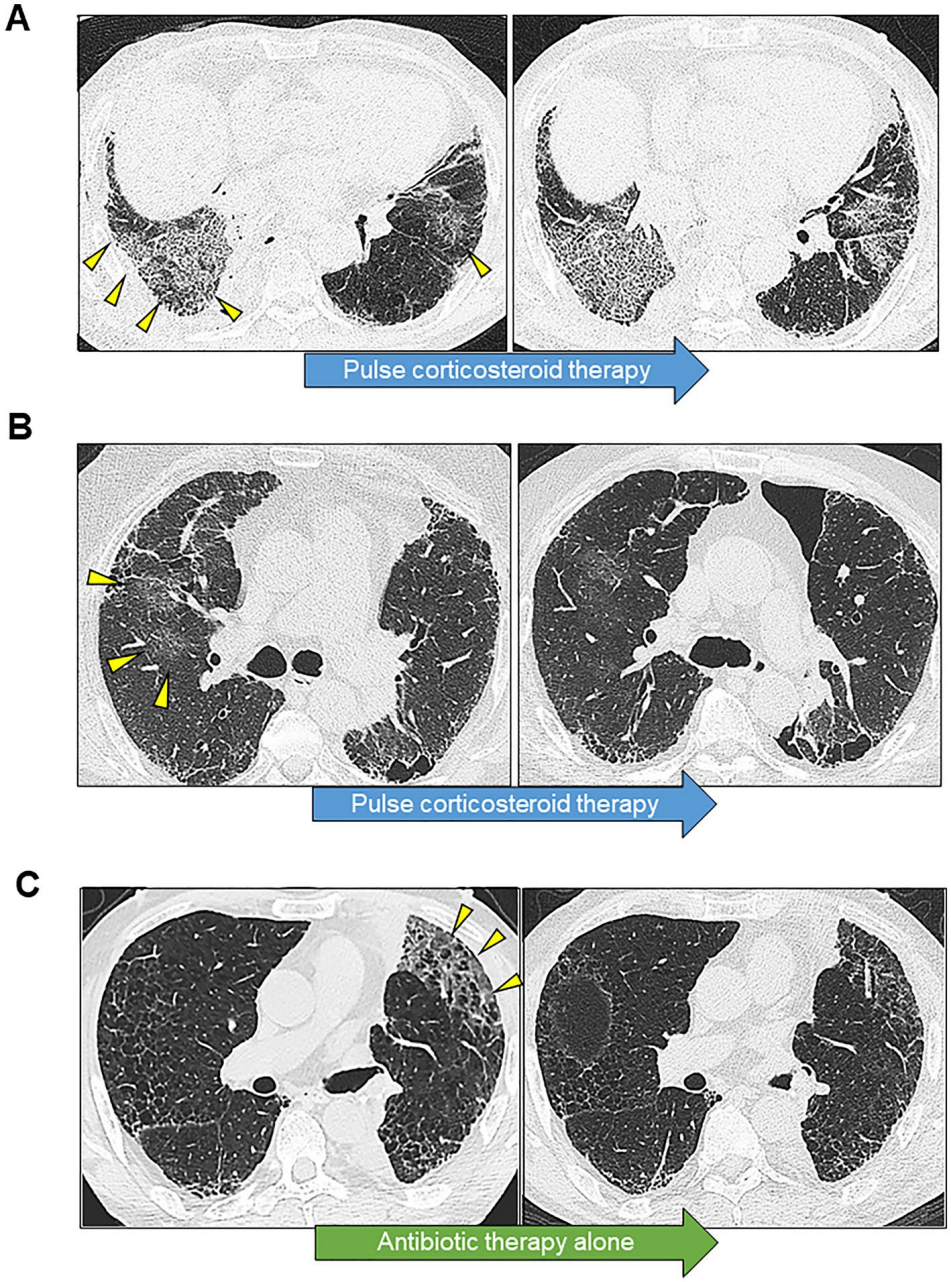
High-resolution computed tomography (HRCT) findings at the time of suspected onset of acute exacerbation (AE) and after treatment in representative cases from each group. **A** Progressive AE with new consolidation and bilateral ground glass opacities on postoperative day (POD) 3 in a patient who had undergone right middle and lower lobe lobectomy (left). Although the patient complained of mild shortness of breath on exertion, the decreasing PaO_2_ was 10.2 mmHg. Despite the administration of pulse corticosteroid therapy, the opacity expanded further (right). The patient died 6 days after the onset of AE. **B** Incipient AE with new ground glass opacities in the right lung on POD 2 in a patient on home oxygen therapy after partial resection of the left lower lobe (left). Although the only reported symptom was mild shortness of breath at rest, the decreasing PaO2 was 15.9 mmHg. The abnormal opacities resolved after one round of pulse corticosteroid therapy and the patient was discharged (right). **C** Indeterminate AE with new peripheral ground glass opacities on POD 9 in a patient who had undergone left lower lobectomy (left). The patient complained of mild shortness of breath on exertion, and there was no decrease in PaO_2_. Pneumonia was diagnosed and the opacity resolved after antibiotic therapy (right)

Among the 93 patients with diagnosed IIP, who underwent surgery for lung cancer, we identified 20 (21.5%) with suspected postoperative AE. These patients were divided into a progressive AE group (*n* = 5, Fig. 2A), an incipient AE group (*n* = 10, Fig. 2B), and an indeterminate AE group (*n* = 5, Fig. 2C). Table 2 shows the patient characteristics of each group. There were no significant differences in predicted forced vital capacity (FVC%pred), predicted forced expiratory ventilation in 1 s, predicted diffusing capacity of the lungs for carbon monoxide (DL_CO_%pred), Krebs von Den Lungen-6 (KL-6), Gender–Age–Physiology staging, PaO_2_/ inspiratory fraction of oxygen (FiO_2_) ratio, HRCT pattern of IIP, pathologic pattern of IIP, surgical procedure, or treatment for IIP among the groups. We also analyzed the risk scoring system for postoperative ILD-AE proposed by the Japanese Association for Chest Surgery [8]. No significant differences in risk score, predicted incidence, or patient risk were observed among the groups. It was suspected that the onset of AE occurred earlier in the incipient AE group than in the progressive AE group, but the difference was not significant (POD 5.2 vs POD 7.4, *P* = 0.335). Patients in the indeterminate AE group tended to be younger than those in the progressive AE group (72.2 vs 64.8, *P* = 0.047). Regarding pathologic staging, patients in the progressive AE group had more advanced lung cancer than those in the indeterminate AE group (*P* = 0.024).

**Table 2:** Comparison of baseline characteristics among patients in the progressive, incipient and indeterminate acute exacerbation groups

	Progressive AE (*n* = 5)	Incipient AE (*n* = 10)	P*	Indeterminate AE (*n* = 5)	P**
Age, years	72.2 ± 4.4	70.5 ± 5.5	0.779	64.8 ± 5.9	0.047
*Pulmonary function*					
FVC%pred (%)	83.5 ± 16.0	96.9 ± 18.8	0.290	97.6 ± 15.9	0.348
DLCO%pred (%)	68.5 ± 13.5	69.8 ± 17.1	0.983	74.8 ± 14.6	0.748
FEV_1_%pred (%)	91.4 ± 12.6	105.4 ± 24.0	0.327	90.3 ± 11.8	0.994
KL-6	925.2 ± 650.7	702.7 ± 298.5	0.499	588.0 ± 127.7	0.362
*GAP staging*					
I/ II	4 (80.0)/ 1 (20.0)	7 (70.0)/ 3 (30.0)	1.000	5 (100)/ 0	1.000
Preoperative P/F ratio	408.3 ± 34.9	386.5 ± 45.6	0.536	404.3 ± 39.9	0.983
*HRCT pattern*					
UIP or probable UIP	5 (100)	8 (80.0)	0.524	4 (80.0)	1.000
*Pathologic pattern of IIP*					
UIP or probable UIP	4 (80.0)	9 (90.0)	0.591	3 (60.0)	0.490
*RS for postoperative AE*^†^					
Score	11.8 ± 2.5	9.8 ± 2.8	0.199	11.0 ± 2.6	0.629
Predicted incidence (%)	15.3 ± 8.4	9.4 ± 5.5	0.121	12.5 ± 7.4	0.589
*Patients’ risk*					
Low/ intermediate	1 (20.0)/4 (80.0)	4 (40.0)/ 6 (60.0)	0.439	2 (40.0)/ 3 (60.0)	0.490
*Pathologic staging*					
I/ II/ III/ IV	0/ 1 (20.0)/ 4 (80.0)/ 0	2 (20.0)/ 5 (50.0)/ 3 (30.0)/ 0	0.242	3 (60.0)/ 1 (20.0)/0/ 1 (20.0)	0.024
*Surgical procedure*					
Lobectomy	5 (100)	9 (90.0)	1.000	5 (100)	1.000
*Treatment for IIP*					
Antifibrotic agents/ NAC	1 (20.0)/ 1 (20.0)	0/ 2 (20.0)	0.670	0/ 0	0.444
POD of suspected AE onset	7.4 ± 5.3	5.2 ± 3.3	0.335	7.4 ± 3.2	1.000

Data are presented as *n* (%) or mean ± SD. *Significance level for comparison between progressive AE and incipient AE groups. ** Significance level for comparison between progressive AE and indeterminate AE groups. AE; acute exacerbation; FVC%pred; percentage of predicted forced vital capacity; DLCO%pred, percentage of predicted diffusing capacity of the lungs for carbon monoxide; FEV_1_%pred, percentage of predicted forced expiratory ventilation in 1 s (%); *GAP staging* Gender-Age-Physiology staging, *PaO*_*2*_ arterial oxygen partial pressure, *FiO*_*2*_ fraction of inspired oxygen, *HRCT* high-resolution computed tomography, *UIP* usual interstitial pneumonia, *RS* risk scoring, *NAC* N-acetylcysteine, *POD* postoperative day. †Based on the postoperative ILD-AE RS system proposed by the Japanese Association for Chest Surgery in reference 8

Supplementary Table 1 shows the patient characteristics, including physical, radiological, and blood test results in the progressive AE and other groups at the time of suspected AE onset. The PaO_2_/FiO_2_ ratio in the progressive AE group was significantly lower than that in the indeterminate AE group (281.5 vs 365.9, *P* = 0.004), but the difference between the progressive AE and incipient AE groups was not significant (281.5 vs 281.5, *P* = 1.000). There were no significant differences in hospital stay, physical findings, number of new parenchymal abnormalities, white blood cell (WBC) count, serum C-reactive protein (CRP) levels, or serum lactate dehydrogenase (LDH) levels among the groups.

### Perioperative mortality and long-term prognosis

**Fig. 3:**
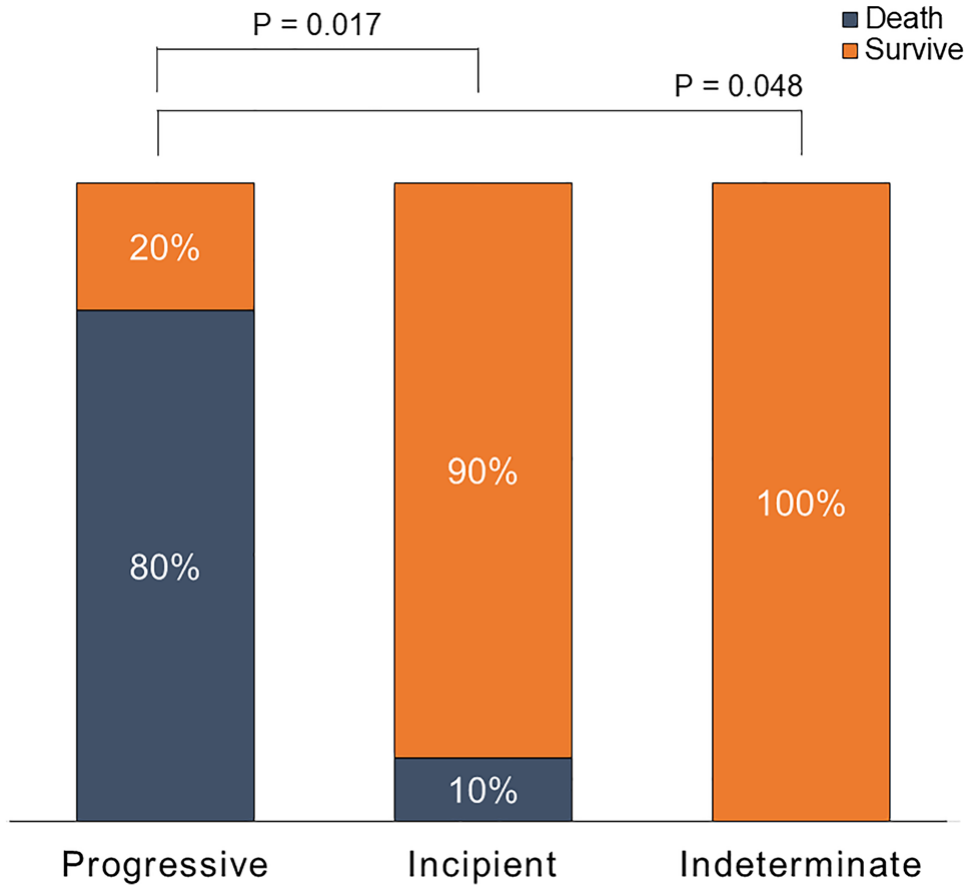
90-day mortality in the progressive AE, incipient AE, and indeterminate AE groups

Figure 3 shows the 90-day mortality in each group. The initial treatment for AE was concurrent pulsed corticosteroid therapy and antibiotic therapy in both the progressive AE and incipient AE groups. Despite receiving the same treatment for AE, the progressive AE group had significantly higher 90-day mortality than the incipient AE group (80.0% vs 10.0%, *P* = 0.017). On the other hand, all patients in the indeterminate AE group received antibiotic therapy alone, with 90-day mortality of zero (80% vs 0%, *P* = 0.048).

**Fig. 4:**
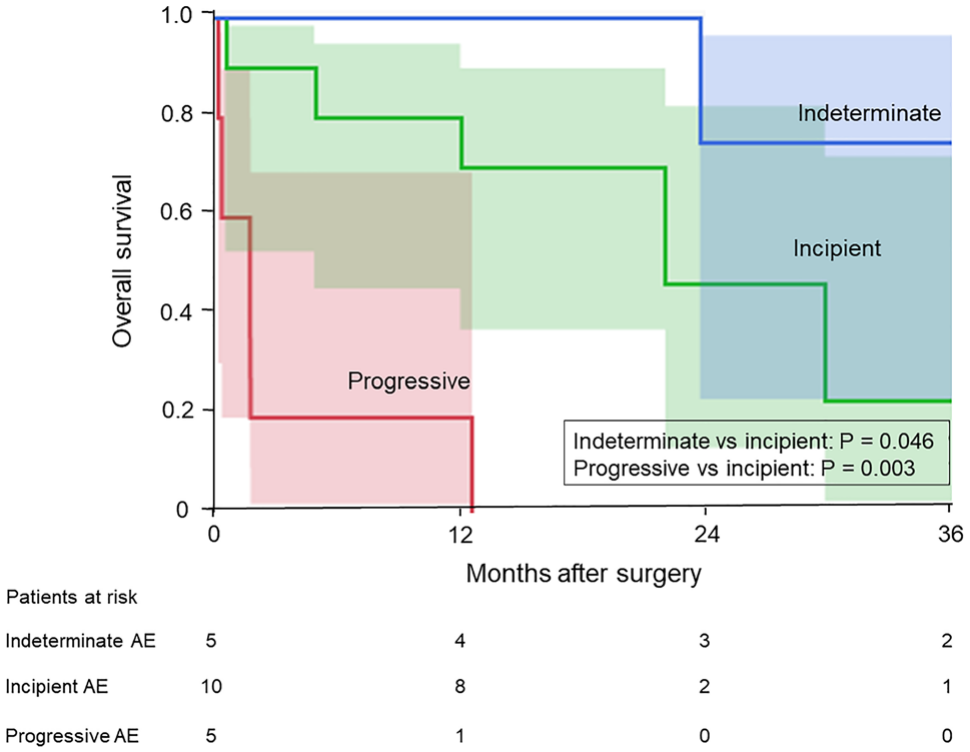
Survival curves for the progressive AE, incipient AE, and indeterminate AE groups

The 3-year survival rates for the progressive AE group, incipient AE group, and indeterminate AE group were 0%, 23.3%, and 75.0%, respectively (progressive vs incipient, *P* = 0.003; incipient vs indeterminate, *P* = 0.046, Fig. 4).

### Univariate and multivariate analyses of factors associated with overall survival

Univariate analysis was performed on data from all patients with suspected postoperative AE. The FVC%pred, DLco%pred, pathologic staging, and classification of AE were identified as factors associated with long-term survival. Multivariate analysis identified the FVC%pred and AE classification as being independently associated with overall survival (Table 3).

**Table 3:** Univariate and multivariate analyses of overall survival for all patients with suspected postoperative acute exacerbation

Categories	Univariate analysis			Multivariate analysis		
	HR	95% CI	*p* value	HR	95% CI	*p* value
*Age (years)*			0.841	–	–	–
< 70	1.00	Reference		–	–	–
≥ 70	1.12	0.36–3.53		–	–	–
*IIP classification*			0.754	**–**	–	–
Others	1.00	Reference		**–**	–	–
IPF	1.39	0.18–11.03		**–**	–	–
*FVC%pred*			0.015			0.020
≥ 80%	1.00	Reference		1.00	Reference	
< 80%	5.47	1.39–21.51		7.37	1.37–39.57	
*DL*_*CO*_*%pred*			0.038			0.320
≥ 80%	1.00	Reference		1.00	Reference	
< 80%	5.05	1.09–23.42		2.67	0.38–18.55	
*Pathologic staging*			0.024			0.687
I, II	1.00	Reference		1.00	Reference	
III, IVA	4.24	1.21–14.78		1.32	0.34–5.14	
*Classification of AE*			0.003			0.031
Indeterminate AE	1.00	Reference		1.00	Reference	
Incipient AE	6.99	0.81–60.22	0.077	3.30	0.33–32.60	0.307
Progressive AE	54.45	4.54–652.75	0.002	25.09	1.62–388.74	0.021

*IIP* idiopathic interstitial pneumonia, *IPF* idiopathic pulmonary fibrosis, *FVC%*pred, percentage of predicted vital capacity, *DL*_*CO*_*%pred* percentage of predicted diffusing capacity of the lungs for carbon monoxide, *AE* acute exacerbation, *HR* hazard ratio, *CI* confidence interval

## Discussion

AE of IIP is still managed according to international working group guidelines [5] and the Japanese Respiratory Society guidelines [6]. However, these guidelines are not unified and differ regarding the spread of radiological abnormalities and the use of arterial blood gas analysis. Furthermore, the same criteria are used for postoperative AE and idiopathic AE, even though these conditions have distinct pathogenic mechanisms and clinical characteristics, including the use of lung resection, general anesthesia, and differential lung ventilation. Accurate and rapid diagnosis and appropriate treatment are essential to improve the survival rate of postoperative AE, which can be fatal, but the current diagnostic criteria may not be suitable for postoperative AE. The purpose of this study was to establish accurate diagnostic criteria and predictors of response to treatment for postoperative AE.

We found that decreasing PaO_2_ and the laterality of new pulmonary abnormalities on HRCT had utility in identifying patients with definitive AE and predicting treatment response. The incipient AE group had a favorable treatment response and a good prognosis, whereas the progressive AE group had high mortality. Bilateral abnormal opacities may represent advanced AE and predict poor treatment response, whereas unilateral opacities may indicate an early stage of AE and predict good treatment response. On the other hand, patients in the indeterminate AE group improved without treatment for AE. Criteria for the diagnosis of postoperative AE are important for deciding on treatment strategies for patients with IIP and lung cancer, because of the possible requirement for intensive care of AE patients with a poor prognosis and to avoid inappropriate treatment for patients who are unlikely to develop AE.

The development of postoperative AE after lung cancer surgery for patients with IIP can be fatal and survivors may have limitations in performing activities of daily living [1]. Sato et al. reported that AE developed within 30 days after pulmonary resection in 9.3% of 1763 patients with ILD, with a mortality of 43.9%. For thoracic surgeons, postoperative AE is the most critical postoperative complication in ILD patients with lung cancer and is challenging to manage. Several studies have identified predictors of postoperative AE, including advanced lung cancer, lack of postoperative steroid use, postoperative episode of inflammation, male sex, KL-6 level, HRCT pattern, predicted vital capacity, history of AE, surgical procedure [2, 9], general anesthesia [10], and perioperative blood transfusion [11]. However, the results of these retrospective studies need to be confirmed for reproducibility. Furthermore, effective prophylaxis against postoperative AE has not been confirmed [1]. The Japanese guidelines state that patients with ILD should not be given preventative drugs preoperatively, apart from antifibrotic agents, and the therapeutic efficacy of prophylactics is limited. [12]

There are no treatments with proven efficacy for AE other than pulse corticosteroid therapy [6]. Postoperative steroid administration may cause secondary complications such as exacerbation of infection and delayed wound healing, and the misdiagnosis of AE must also be avoided. Accordingly, accurate diagnosis, and appropriate treatment of acute postoperative exacerbations are of the utmost importance. The common criteria in the international [5] and Japanese criteria [6] for the diagnosis of AE-ILD are worsening dyspnea and the appearance of new parenchymal abnormalities. However, patients who have undergone pulmonary resection may experience breathlessness and dyspnea as a result of their decreased lung capacity, even in a normal postoperative course. New pulmonary abnormalities can appear for a variety of reasons, including poor aeration around the staple line or postoperative pneumonia. Elevated WBC, CRP, and LDH, defined as reference findings [6] are also seen in the postoperative course, related to surgical stress. Accordingly, a decrease in PaO2, which is included only in the Japanese diagnostic criteria, may be the most useful way of definitively diagnosing postoperative AE in patients with lung cancer. PaO_2_ and the PaO_2_/FiO_2_ ratio are also used as diagnostic criteria for acute lung injury (ALI) and acute respiratory distress syndrome (ARDS) [13]. The similar etiology and pathological findings between AE and ALI/ARDS [5] support the hypothesis that arterial blood gas findings can contribute to confirming the diagnosis of postoperative AE. The progressive AE and incipient AE groups in our study had a mean PaO_2_/FiO_2_ ratio < 300 mmHg, which is diagnostic of ALI [13] at the onset of AE, while the indeterminate AE group had a mean PaO_2_/FiO_2_ ratio > 300 mmHg.

Several prognostic factors for idiopathic AE have been reported, including PaO_2_/FiO_2_ ratio < 250, serum CRP level ≥ 5.5, and diffuse IPF pattern on HRCT [14, 15]; however, prognostic factors for postoperative AE have yet to be reported. Our data demonstrate significantly worse 90-day mortality for patients with bilateral pulmonary abnormalities on HRCT than for those with unilateral pulmonary abnormalities. Although the development of bilateral pulmonary abnormalities is a diagnostic criterion in the international guidelines for the diagnosis of AE, [5] our data indicate that bilateral pulmonary abnormalities are also an indicator of treatment-resistant progressive disease in patients with postoperative AE. In contrast, patients with unilateral pulmonary abnormalities had a favorable response to treatment and good prognosis. The perioperative period provides an opportunity for close observation of radiographic and physical findings, which may help identify the early onset of AE. There has been recent discussion regarding sub-AE and the staging of AE [5], with further accumulation of evidence awaited. In contrast to patients with a chronic course of IIP, lung cancer patients with postoperative IIP have decreased lung volume. Accordingly, rapid treatment may be necessary before parenchymal abnormalities expand bilaterally, which is associated with a greater risk of mortality for patients with postoperative AE-IIP.

Based on the results of this study, we propose the following diagnostic and treatment strategies for postoperative AE-IIP in patients with lung cancer: Progressive AE should be diagnosed in patients with new bilateral radiologic abnormalities and decreasing PaO_2_ ≥ 10 mmHg. Immediate intensive care for AE is required to improve the poor prognosis of patients with progressive AE. Incipient AE should be diagnosed in patients with new unilateral radiologic abnormalities and decreasing PaO_2_ ≥ 10 mmHg. Immediate treatment for AE in this patient group can lead to a favorable prognosis. Indeterminate AE should be diagnosed in patients with any new radiologic abnormality and decreasing PaO_2_ < 10 mmHg, for whom the treatment of other diseases, such as pneumonia, takes priority. Patients in the indeterminate AE group may have other clinical conditions that require further investigation.

The present study had limitations and biases. First, it was a retrospective and single-institute study, and the findings may not be generalizable to other patient populations. Second, differences in the age and staging of lung cancer between the progressive AE group and other groups may have affected the postoperative course. Finally, changes in surgical technique, surgical methods, or anesthetic agents over the study period may have biased the incidence of postoperative complications. Further prospective studies by multiple institutes are required to validate the findings of the present study.

## Conclusions

In patients with lung cancer and IIP, decreasing PaO_2_ and progressive parenchymal abnormalities on HRCT have utility in the diagnosis of postoperative AE-IIP and the prediction of prognosis. These criteria may contribute to the development of a rapid and accurate treatment strategy for postoperative AE-IIP in lung cancer patients.

### Supplementary Information

Below is the link to the electronic supplementary material.

Supplementary file1 (DOCX 23 KB)

## Abbreviations

ILD: Interstitial lung disease
AE: Acute exacerbation
IIP: Idiopathic interstitial pneumonia
IPF: Idiopathic pulmonary fibrosis
PaO_2_: Partial pressure of arterial oxygen
POD: Postoperative day
BGA: Arterial blood gas analysis
HRCT: High-resolution computed tomography
FVC%pred: Percentage of predicted forced vital capacity
DL_CO_%pred: Percentage of predicted diffusing capacity of the lungs for carbon monoxide
SD: Standard deviation
OS: Overall survival
KL-6: Krebs von Den Lungen-6
FiO_2_: Inspiratory fraction of oxygen
WBC: White blood cells
CRP: C-reacted protein
LDH: Lactate dehydrogenase
ALI: Acute lung injury
ARDS: Acute respiratory distress syndrome
