# Supplementary material for: Impact of partial pressure of arterial oxygen and radiologic findings on postoperative acute exacerbation of idiopathic interstitial pneumonia in patients with lung cancer
Source: Surg Today. 2023 Jun 6;54(2):122–9. doi: 10.1007/s00595-023-02711-y (PMC10803386; doi:10.1007/s00595-023-02711-y)
Supplement: Supplementary file 1 — Supplementary file1 (DOCX 23 KB) [file 595_2023_2711_MOESM1_ESM.docx]

Supplementary Table 1. Comparison of patient characteristics among patients in the progressive, incipient, and indeterminate acute exacerbation groups at the onset of suspected acute exacerbation.

|  | **Progressive AE**  (n = 5) | **Incipient AE**  (n = 10) | **P*** | **Indeterminate AE**  (n = 5) | **P^**^** |
| --- | --- | --- | --- | --- | --- |
| **Breathlessness** |  |  |  |  |  |
| On exertion/ at rest | 2 (40.0)/ 3 (60.0) | 6 (60.0)/ 2 (20.0) | 0.324 | 3 (60.0)/ 2 (40.0) | 0.167 |
| **New alveolar abnormality** |  |  |  |  |  |
| GGO/ consolidation/ both | 3 (60.0)/ 0/ 1 (40.0) | 9 (90.0)/ 0 / 1 (10.0) | 0.242 | 3 (60.0)/ 1 (20.0)/ 1 (20.0) | 1.000 |
| Number | 4.6 ± 1.9 | 3.0 ± 1.8 | 0.455 | 4.2 ± 4.1 | 0.850 |
| Unilateral/healthy side | 0 | 10 (100)/ 7 (70.0) | - | 2 (40.0)/ 1 (20.0) | - |
| Bilateral | 5 (100) | 0 | - | 3 (60.0) | 0.444 |
| **Arterial blood gas analysis** |  |  |  |  |  |
| PaO_2_/FiO_2_ ratio | 281.5 ± 43.0 | 281.5 ± 40.4 | 1.000 | 365.9 ± 43.1 | 0.015 |
| **Blood test** |  |  |  |  |  |
| WBC (10^3^/µL) | 12.2 ± 3.7 | 10.1 ± 3.3 | 0.405 | 10.7 ± 1.5 | 0.435 |
| CRP (mg/dL) | 9.7 ± 6.3 | 10.3 ± 6.7 | 0.978 | 5.1 ± 3.3 | 0.183 |
| LDH (IU/L) | 264.2 ± 23.4 | 250 ± 35.6 | 0.614 | 250.4 ± 19.7 | 0.343 |
| Data are presented as n (%) or mean ± SD. * Significance level for comparison between progressive AE and incipient AE groups. ** Significance level for comparison between progressive AE and indeterminate AE groups. GGO, ground glass opacity; PaO_2_, partial pressure of atrial oxygen; FiO_2_, fractions of inspired oxygen; WBC, white blood cells; CRP, C-reactive protein; LDH, lactate dehydrogenase | | | | | |
